# NEIL2 Protects against Oxidative DNA Damage Induced by Sidestream Smoke in Human Cells

**DOI:** 10.1371/journal.pone.0090261

**Published:** 2014-03-03

**Authors:** Altaf H. Sarker, Arpita Chatterjee, Monique Williams, Sabrina Lin, Christopher Havel, Peyton Jacob III, Istvan Boldogh, Tapas K. Hazra, Prudence Talbot, Bo Hang

**Affiliations:** 1 Department of Cancer & DNA Damage Responses, Life Sciences Division, Lawrence Berkeley National Laboratory, Berkeley, California, United States of America; 2 Division of Pulmonary and Critical Care Medicine, University of Texas Medical Branch, Galveston, Texas, United States of America; 3 Department of Cell Biology & Neuroscience, University of California Riverside, Riverside, California, United States of America; 4 Department of Medicine, University of California San Francisco, San Francisco General Hospital Medical Center, San Francisco, California, United States of America; Roswell Park Cancer Institute, United States of America

## Abstract

Secondhand smoke (SHS) is a confirmed lung carcinogen that introduces thousands of toxic chemicals into the lungs. SHS contains chemicals that have been implicated in causing oxidative DNA damage in the airway epithelium. Although DNA repair is considered a key defensive mechanism against various environmental attacks, such as cigarette smoking, the associations of individual repair enzymes with susceptibility to lung cancer are largely unknown. This study investigated the role of NEIL2, a DNA glycosylase excising oxidative base lesions, in human lung cells treated with sidestream smoke (SSS), the main component of SHS. To do so, we generated NEIL2 knockdown cells using siRNA-technology and exposed them to SSS-laden medium. Representative SSS chemical compounds in the medium were analyzed by mass spectrometry. An increased production of reactive oxygen species (ROS) in SSS-exposed cells was detected through the fluorescent detection and the induction of HIF-1α. The long amplicon–quantitative PCR (LA-QPCR) assay detected significant dose-dependent increases of oxidative DNA damage in the *HPRT* gene of cultured human pulmonary fibroblasts (hPF) and BEAS-2B epithelial cells exposed to SSS for 24 h. These data suggest that SSS exposure increased oxidative stress, which could contribute to SSS-mediated toxicity. siRNA knockdown of NEIL2 in hPF and HEK 293 cells exposed to SSS for 24 h resulted in significantly more oxidative DNA damage in *HPRT* and *POLB* than in cells with control siRNA. Taken together, our data strongly suggest that decreased repair of oxidative DNA base lesions due to an impaired NEIL2 expression in non-smokers exposed to SSS would lead to accumulation of mutations in genomic DNA of lung cells over time, thus contributing to the onset of SSS-induced lung cancer.

## Introduction

Secondhand smoke (SHS, also called environmental tobacco smoke, ETS) is a mixture of ∼85% of sidestream smoke (SSS, the smoke coming off the end a smoldering cigarette) and ∼15% of exhaled mainstream smoke (MSS). Exposure to SHS remains widespread in many countries, and affects a large population of adult and young non-smokers worldwide. SHS exposure primarily takes place in homes and workplaces, as well as common public locations such as restaurants, bars, and casinos. Based on the recent National Health and Nutrition Examination Survey data, an estimated 88 million nonsmokers and nearly half of the children between ages 3–11 in the U.S. were exposed to SHS between 2007–2008 [1]. Such data highlight the fact that children are at risk for SHS exposure.

Based on the U.S. Surgeon General, there is no risk-free level of exposure to SHS; even brief or small amounts of exposure can be harmful to human health [2]. In children, the most common symptoms found after SHS exposure are those associated with the respiratory system, including asthma and infections, as well as decreased lung function. Also SHS increases the risk of sudden infant death syndrome (SIDS). In adult nonsmokers exposed to SHS, there is an increased risk for lung cancer [3], [4]. SHS exposure causes an estimated 3,400 lung cancer deaths annually among adult nonsmokers in the U.S. [5]. The U.S. Surgeon General estimates that living with a smoker increases a nonsmoker's chances of developing lung cancer by 20–30% [6]. Contact with SHS has also been implicated in the risk increase of other types of cancers, such as nasal sinus cavity cancer, nasopharyngeal cancer, breast cancer, leukemia, and brain tumors in children [6]. SHS exposure is also associated with cardiovascular diseases, such as coronary artery disease.

Although the above findings provide considerable support for the association of SHS with various human diseases, the molecular mechanisms underlying the relationship between SHS exposure and pulmonary diseases are still poorly understood. Cigarette smoke is a mixture of gases and fine particles that includes more than 7000 chemicals, including hundreds of toxic compounds and about 70 known carcinogens [7], [8]. SHS also contains thousands of chemicals, many of which are oxidants and contribute to oxidative stress via induction of reactive oxygen species (ROS) and pro-inflammatory mediators. Such effects are particularly significant in the lung, as it is the organ that is directly exposed to the chemicals in SHS. Bronchial epithelial cells are reported to be exposed to oxidative and carcinogenic compounds that can cause damage to molecules such as DNA [9]. The mutations that are caused by oxidative base lesions are associated with many types of human disorders, particularly cancer [10]. ROS-induced oxidation of DNA is normally complex, including a variety of DNA base modifications, strand breaks, and ring opening of the modified base, all of which are expected to be contributors to the pathophysiology of SHS.

Oxidized DNA bases can cause either point mutations or block transcription of an essential gene. To counteract the deleterious effect of these lesions, cells have developed DNA repair mechanisms for their removal. The efficiency of such repair was frequently found to be low in cells of patients with cancers, such as lung cancer [11]. Therefore, deficiency in DNA repair could play an important role in SHS-induced mutagenesis and carcinogenesis in humans. Oxidized bases are primarily repaired by the base excision repair (BER) pathway [12], which is initiated by a DNA glycosylase that excises the damaged base. So far five DNA glycosylases have been identified that act on oxidized bases with distinct but overlapping substrate specificities. Of these enzymes, only 8-oxoguanine DNA glycosylase (OGG1) has been implicated in the development of lung cancer and cancers from other organs [13]–[15]. Recently, we found that a defective function of another enzyme in this group, endonuclease VIII-like 2 (NEIL2), can lead to a significant increase in mutation frequency in cultured mammalian lung cells [16]. This enzyme belongs to a class of DNA glycosylases homologous to the bacterial Fpg/Nei family and mainly excises oxidation products formed on cytosine, preferring 5-hydroxyuracil (5-OHU) and 5-hydroxycytosine (5-OHC) [17], [18]. We also identified a novel polymorphic NEIL2 variant R257L that possesses reduced total BER activity towards oxidized bases and is present at significantly higher levels in lung cancer patients than in controls in certain human populations [16]. Although a correlative effect of genotype and smoking was not observed, the unusually high percentage of non-smokers with R257L having lung cancer was thought to have a linkage to their exposure to SHS. In any case, these results suggest that NEIL2 could be an important DNA repair enzyme that plays a role in the defense against oxidative DNA damage. This study investigated whether NEIL2 deficiency would result in the accumulation of oxidative base damage in human pulmonary cells following exposure to SSS, which constitutes approximately 85% of SHS.

## Materials and Methods

### Materials

d0-, d8-nicotelline, d0-, d9-cotinine and d0-, d4-pseudooxynicotine (4-(methylamino)-1-(3-pyridinyl)-1-butanone) were synthesized in the University of California, San Francisco (UCSF) Clinical Pharmacology Laboratory (manuscript in preparation). d0- and d3-NNA, d0- and d4-NNK, d0- and d4-NNN, d0- and d4-bipyridine (2,3′-bipyridyl, 2,3′-dipyridyl), and *N*-formylnornicotine (*N*-formyl-2-[3-pyridyl] pyrrolidine) were procured from Toronto Research Chemicals, Ontario, Canada. Pentafluorophenylhydrazine (PFPH, 97%) was obtained from Sigma-Aldrich, Saint Louis, MO. All other reagents and HPLC grade solvents were from Fisher Scientific (Waltham, MA).

### Immunohistochemical staining of NEIL2

The levels of NEIL2 were analyzed in a lung adenocarcinoma tissue microarray (33 cases and normal controls) (grade I–III, US Biomax, Rockville, MD) by immunohistochemistry. Paraffin sections were deparafinized at 55°C for 1 h using xylene and various concentrations of ethanol, and finally rehydrated with water. The sections were incubated with sodium citrate buffer at 95°C for 20 min for antigen retrieval and subsequently incubated with affinity purified IgG (500 µg/mL) from non-immune serum to block the non-specific binding sites. The sections were then incubated with primary antibody (NEIL2 antibody, 1∶100, Santa Cruz) overnight at 4°C. A fluorescein-conjugated affinity purified secondary antibody (Santa Cruz Biotechnology, Inc.) was then applied to sections to visualize primary antibody binding. Antibody exposure and washing were carried out in phosphate buffered saline (PBS)-T containing 0.5% BSA. Nuclei were stained for 15 min with DAPI (4,6-diamidino-2-phenylindole dihydrochloride; 10 ng/ml). Sections were then mounted in anti-fade medium (Dako Inc. Carpinteria, CA). Confocal microscopy was performed on a Zeiss LSM510 META System using the 488 nm excitation of FITC and 358 nm for DAPI, combined with appropriate dichroic mirrors and emission band filters to discriminate between green and blue fluorescence. Images were captured at a magnification of 60X (oil immersion objective; numerical aperture 1.4). Fluorescence intensities of a minimum of 100 nuclei per section were determined using MetaMorph software Version 4.6r9 (Universal Imaging Corp) as described previously [19].

### Preparation of SSS solution

A commercial brand of popular cigarettes was purchased from a local retail dealer and used to make SSS solutions with a method described previously in detail [20], [21]. Smoke solutions were generated using a University of Kentucky smoking machine that took a 2.2 second puff of mainstream smoke every min. SSS was continuously collected for 20 min (the time required to take 20 mainstream puffs) from the burning end of a cigarette and passed through 10 ml of DMEM culture medium. Concentrations of smoke solutions were measured in puff equivalents (PE) (1 PE =  the amount of SSS collected in 1 min that dissolves in one ml of medium). SSS solutions were made at concentrations of 2 PE. Once completed, smoke solutions were filtered through a 0.2 micron filter, aliquoted into Eppendorf tubes, and stored in −80°C freezer.

### Chemical analysis of SSS samples

Aliquots of media (0.8–1 mL) were stored at −20°C until analyzed. The SSS-laden DMEM samples were analyzed by gas chromatography – mass spectrometry (GC-MS) for 3-ethenylpyridine and myosmine (manuscript in preparation), and other components by liquid chromatography-tandem mass spectrometry (LC-MS/MS) as previously described [22], [23].

### Cell culture

Human BEAS-2B lung epithelial cells were from the American Type Culture Collection (ATCC, Manassas, VA). Human pulmonary fibroblasts (hPF, ScienCell, Carlsbad, CA) and human embryonic kidney 293 (HEK293) cells were grown at ambient oxygen levels and 10% CO_2_ in DMEM supplemented with 10% fetal calf serum before treatment. Our laboratories have been using HEK293 cells for many transfection-based experiments, therefore, this cell line was used along with the two pulmonary cell lines. Cells were treated with various concentrations of SSS for 24 h before collecting by trypsinization.

### SSS induces ROS production in human pulmonary fibroblasts (hPF)

To demonstrate production of ROS in SSS-treated hPF, cells were preloaded with 5 µM MitoSOX™ Red (Life technologies, Grand island, NY) for 10 min at 37°C and 5% CO_2_. They were then washed twice with PBS containing Ca^2+^ and Mg^2+^. After washing, cells were incubated and imaged in a BioStation IM in either control medium (complete fibroblast medium) or medium containing 0.03PE of SSS solution. Phase contrast and fluorescent images were taken at 10X magnification every 4 min for 600 min (10 h). Fluorescent intensity was analyzed in time-lapse images using a video bioinformatics protocol developed with CL-Quant software (DRVision Technology LLC, Bellevue, WA). Two experiments were performed with 4–5 videos per group in each experiment. Phase contrast and fluorescent images were merged using CL-Quant software.

### Induction of hypoxia-inducible factor-1α (HIF-1α)

BEAS-2B cells were grown in DMEM supplemented with 10% FBS in a six well plate (5×10^5^ cells/well) the day before starting SSS treatment. The following day, cells were treated with SSS solution at the desired dose for 24 h, after which, cells were collected by trypsinization. Whole cell extracts were prepared and separated by SDS-PAGE, and Western blot analysis was carried out using anti-HIF-1α (Bethyl Laboratories, Montgomery, TX), with GAPDH (Millipore, Temecula CA) being the loading control. The band intensity was quantitated with ImageQuant (Molecular Dynamics). The data were plotted as histograms with fold increase as Y-axis, which was calculated comparing the values of exposed samples with the control.

### siRNA-mediated knockdown of NEIL2

hPF or BEAS-2B cells were transfected with siRNA against 3′-UTR of NEIL2 (sense: 5′-GAAUGAACCUAGAGCGGUGUU; anti-sense: phos-CACCGCUCUAGGUUCAUUCUU) or with a nonspecific siRNA (sense: 5′-GAAAAGGAGGAUGCUAAACGU-3′; anti-sense: phos-GUUUAGCAUCCUCCUUUUCUU) using Lipofectamine™RNAiMAX (Invitrogen). Briefly, 1×10^5^ cells were seeded per well in a six-well dish the day before transfection. The cells were washed with PBS in the following day and 2 ml OPTI-MEM was then added to the wells. 100 pmol siRNA and 5 µL RNAiMAX were used for transfection following the manufacturer's protocol. The same transfection step was repeated the next day followed by 48 h of incubation with addition of normal growth medium. The cells were then ready for the treatment with SSS solution at 37°C for 24 h. The knockdown efficiency of NEIL2 was examined by quantitative real-time (qRT)-PCR using a NEIL2 specific TaqMan probe (Applied Biosystems). Knockdown of NEIL2 following expression of a NEIL2-FLAG construct in HEK293 cell was the same as described earlier [16].

### LA-QPCR assay

After exposure to SSS-treated medium (0.1, 0.2 and 0.4 PE dilutions) at 37°C for 24 h as described above, the cells were harvested for genomic DNA extraction using the Qiagen Genomic-tip 20/G kit (Qiagen, Valencia, CA) per the manufacturer's instructions. This kit minimizes DNA oxidation during the isolation step and has been used previously for LA-QPCR assays [16]. After quantification equal amounts of genomic DNA were digested with two *E. coli* BER enzymes, Fpg (Trevigen, Gaithersburg, MD) and Nei (provided from Dr. Tapas lab), which are able to remove a variety of oxidized purine and pyrimidine oxidized DNA bases and induce strand breaks by cleaving the phosphodiester bond with their associated AP lyase activity.

To examine the formation of oxidative DNA damage in the human gene, *hypoxanthine phosphoribosyl transferase 1* (*HPRT*), the LA-QPCR assay was performed in SSS-exposed human cells as described previously [16], [22], [24]. LongAmp Taq DNA polymerase (New England Biolabs, Beverly, MA) was used to amplify a 10.4 kb region of *HPRT* in human genomic DNA using the following primers: 5′-TGG GAT TAC ACG TGT GAA CCA ACC-3′ and 5′-GCT CTA CCC TCT CCT CTA CCG TCC-3′. Preliminary assays were carried out to ensure the linearity of PCR amplification with respect to the number of cycles and DNA concentration. Since amplification of a small region would be relatively independent of oxidative DNA damage (low probability), a small DNA fragment for *HPRT* (250 bp) was also amplified for normalization of the data obtained with the large fragments using the following primers: 5′-TGC TCG AGATGT GAT GAA GG-3′ and 5′-CTG CAT TGT TTT GCC AGT GT-3′. All of the amplified products were resolved and visualized using agarose gel electrophoresis and quantitated with an ImageQuant (Molecular Dynamics) or Varsadoc (Bio-Rad) system. The data were plotted as histograms with relative amplification as Y-axis, which was calculated comparing the values of exposed samples with the control.

### Statistical analysis

Data are expressed as means ± SD of 3 experiments. Comparison between the treatments was made by p value determination using Student's t-test. A p value of <0.05 was considered to be statistically significant.

## Results

### NEIL2 expression in lung cancer tissue sections

**Figure 1:**
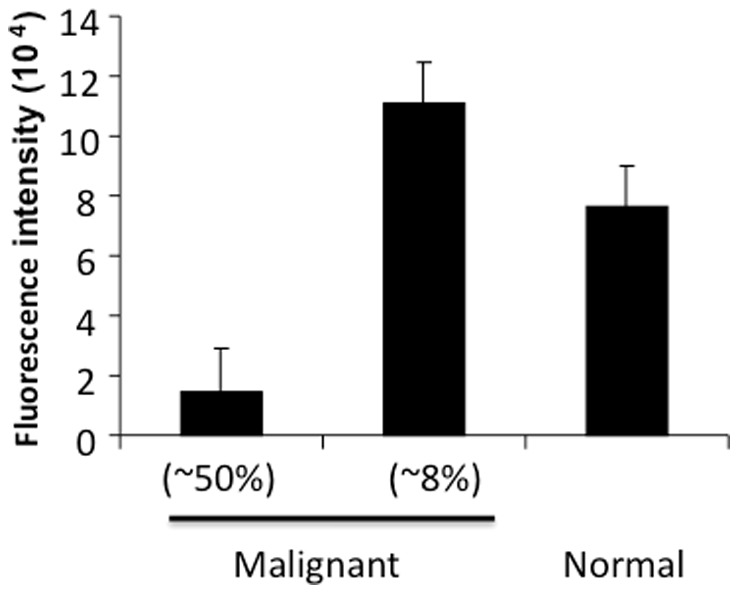
NEIL2 level is low in lung cancer tissues. Tissue NEIL2 level is low in about 50% and high in about 8% of lung cancer cases compared to normal lung tissue, as measured by immunohistochemistry.

The levels of NEIL2 were analyzed in a lung adenocarcinoma tissue microarray (33 cases and normal controls) by immunohistochemistry using affinity purified NEIL2 antibody. NEIL2 protein levels were extremely low in 50% of cancer tissues examined and higher than normal in only 8% cancer cases (**Fig. 1**).

### Measurement of SSS components in medium

SSS solutions were prepared as previously described [20], [21] and then analyzed for chemical biomarkers. Concentrations of various chemical components in the SSS-laden medium were determined using GC-MS and LC-MS/MS. to provide a measure of the amount of SSS present. This served as a quality assurance and quality control measure to ensure the dose and composition of the common chemicals in the medium, which is crucial for the comparison of SHS-induced effects found in our studies. **Table 1** summarizes the concentrations of compounds (ng/ml) detected and quantified in various DMEM samples and the limit of quantification (LOQ) for the analytical methods. The level of SSS exposure was shown in **Table 1** by the levels of nicotine, its main oxidation products, cotinine and myosmine, and other SHS alkaloids in the sample. As shown in **Table 1**, concentrations of all analytes were below limits of detection in DMEM only samples. As for DMEM exposed to blank, only 3-ethenylpyridine was detected but at very low concentrations compared to the level in the SSS-DMEM sample. As expected, **Table 1** shows that nicotine was the major compound of all the SSS components tested in this study. The SSS samples also contained considerable amounts of 3-ethenylpyridine and myosmine, the two commonly measured indicators of SHS.

**Table 1:** Concentrations of SSS constituents in DMEM medium (ng/mL)

Sample name	Nicotine LOQ = 3.05	Cotinine LOQ = 2.74	Nicotelline LOQ = 9.1×10^−2^	3-Ethenyl pyridine LOQ = 3.05×10^−1^	N-Formyl-nornicotine LOQ = 9.14×10^−1^	Myosmine LOQ = 9.14×10^−1^	2,3′-Bipyridine LOQ = 9.14×10^−1^
DMEM Only	BLOQ	BLOQ	BLOQ	BLOQ	BLOQ	BLOQ	BLOQ
Blank	BLOQ	BLOQ	BLOQ	3.27×10^1^	BLOQ	BLOQ	BLOQ
SSS in DMEM	5.759×10^3^	2.63×10^1^	1.96×10^1^	4.048×10^3^	4.39×10^1^	2.26×10^2^	6.88×10^1^

Abbreviations: LOQ: limit of quantification; BLOQ, Below the Limit Of Quantification.

### SSS induces ROS production and increases levels of HIF-1α in hPF

**Figure 2:**
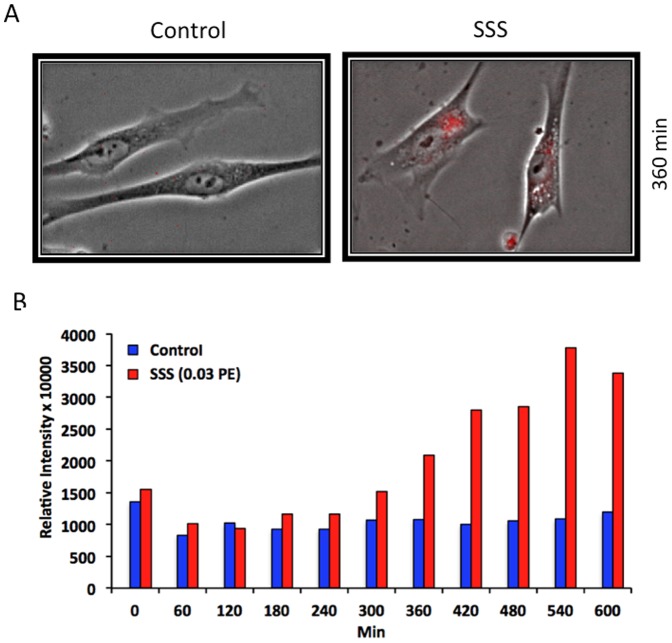
SSS increased ROS production in hPF. Cells were preloaded with MitoSOX™ Red to visualize ROS production. (A) Control cells imaged at 360 min did not have red fluorescence. (B) At 360 min, SS smoke treated cells had red fluorescence indicative of ROS production. (C) Bar graph showing increased intensity of fluorescence in treated cells, but not in control cells, over 600 min. In Figure 2C, data are the means of two experiments; the control and treated groups had 84 and 99 cells, respectively.

hPF, preloaded with MitoSOX™ Red, were incubated with SSS solutions (0.03 PE) for 600 min (10 h) and imaged in a BioStation IM at 4 min intervals. MitoSOX™ Red is a fluorescent reagent that is targeted to mitochondria in live cells. When MitoSOX™ Red is oxidized by superoxide, it fluoresces red. At about 300–360 min of incubation, ROS intensity increased in the SSS-treated cells relative to the control cells (**Fig. 2A–2C**). ROS production remained elevated for the duration of the incubation period. Cells with elevated ROS also had more vesicles in their cytoplasm than the control cells (**Fig. 2B**).

We also examined whether the doses of SSS used in this study increased HIF-1α levels, a marker for ROS production [25]. As shown in **Fig. S1A**, HIF-1α expression was measured in BEAS-2B cells exposed to SSS using Western blotting. SSS at 0.2 and 0.4 PE efficiently induced the expression of HIF-1***α***, whereas the levels of GAPDH in the same cells did not show any increase. It has been reported previously that ROS resulting from H_2_O_2_ induces HIF-1α expression [25]. As a positive control, **Fig. S1B** shows that the expression of the protein was increased when the cells were treated with 1 mM H_2_O_2_ for 60 min.

### Induction of oxidative DNA damage by SSS in the *HPRT* gene of normal human lung cells

The level of oxidative base damage in the *HPRT* gene was measured by the LA-QPCR assay as previously described [16], [22], [24]. This assay is highly sensitive to oxidative DNA damage in several specific mammalian genes when coupled with the use of specific DNA repair enzymes which excise oxidized bases. The *HPRT* gene is a widely used biomarker for detection of DNA damage and mutations in human cells. Two bacterial DNA glycosylases (Fpg and Nei) which excise damaged (oxidized) bases were used to detect oxidative damage in the long (10.4 kB) amplicon. A short (250 bp) amplicon, which was not likely to have oxidative damage, was included for normalization for amplification. A control LA-QPCR assay was performed with or without addition of Fpg/Nei, in which both hPF and BEAS-2B cells were treated with SSS (0.4 PE) for 24 h (**Fig. S2**).

**Figure 3:**
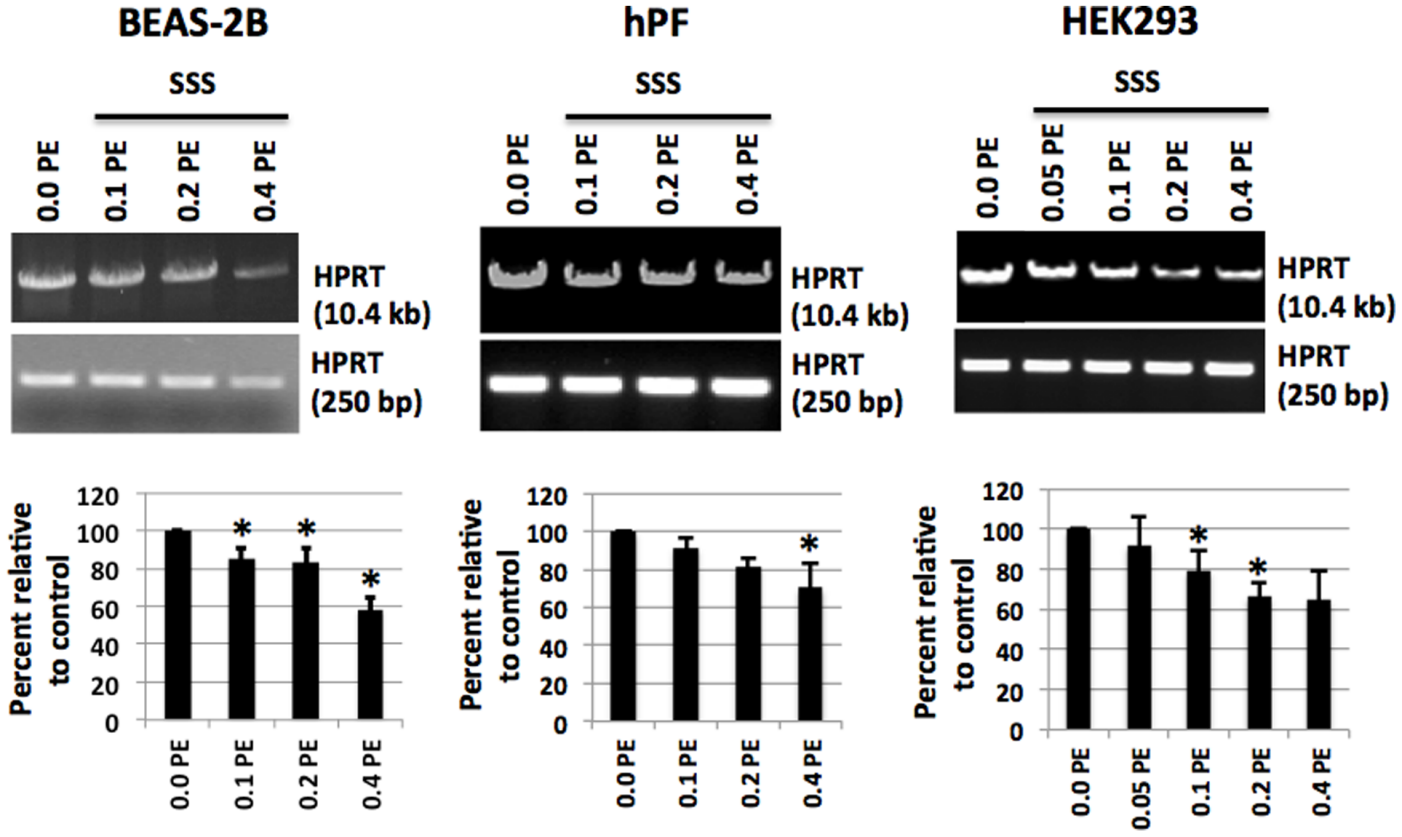
Increased oxidative DNA damage in various human cells. Following exposure to a series of dilutions of SSS–laden DMEM for 24 h, DNA isolation and digestion with Fpg and Nei followed by qPCR for amplification of *HPRT* large and a short fragment (LA-QPCR assay). Three cell lines, HEK293, hPF and BEAS-2B, all exhibited a dose-response relationship between levels of oxidative DNA damage and the SSS doses used in the LA-QPCR assay. PE: puff equivalents (the smoke from one puff in 1 ml of medium).

SSS exposure caused a dose-dependent decrease in the level of the 10.4 kb fragment of the *HPRT* gene in HEK293 cells, hPF cells, and BEAS-2B cells after 24 h exposure (**Fig. 3A**). Compared with blank samples (lane 1, 0.0 PE), the decrease in percentage was between 60–70% for the highest SSS concentration (0.4 PE). The intensity of the short *HPRT* fragment (250 bp), which was used to normalize the large fragment, remained nearly unchanged for all cell types (**Fig. 3**). These data indicate that SSS exposure causes increased levels of oxidative DNA damage in each cell type over the concentration range tested. This information was essential for the experiments performed below which test the effect of NEIL2 knockdown on the levels of oxidative DNA damage in the same cell lines.

### Effects of NEIL2 deficiency on level of oxidative DNA damage in cells exposed to SSS

**Figure 4:**
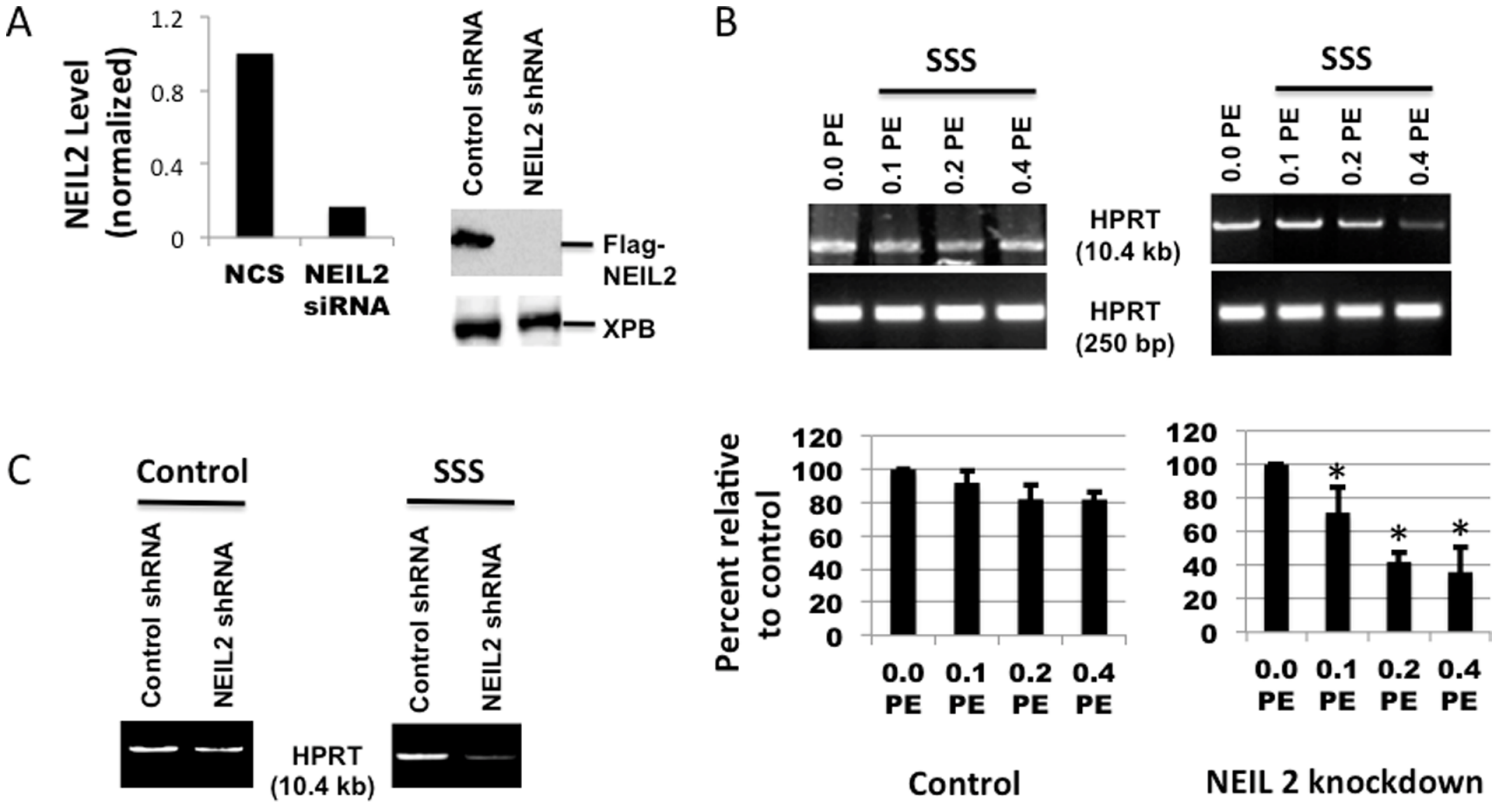
Effect of NEIL2 knockdown on induction of oxidative DNA damage by exposure to SSS for 24*HPRT* gene using LA-QPCR. (A) left: NEIL2 knockdown from hPF was tested by qRT-PCR; right: Western blots showing shRNA-mediated knockdown of NEIL2 in HEK293 cells expressing Flag-NEIL2. (B) Cultured hPF with NEIL2 knockdown were treated with 3 doses of SSS extract as compared to the control followed by DNA isolation and digestion with Fpg and Nei as described in Materials and Methods. The DNA was used as template for *HPRT* large and short fragment amplification by LA-QPCR assay. The histogram depicts the mean % of DNA tail ± SEM of 3 to 4 independent experiments. The symbol * means that the differences between the Control and NEIL 2 knockdown were statistically significant (P<0.05); (C) HEK293 cells with NEIL2 knockdown without (left) or with (right) SSS treatment followed by *HPRT* large fragment amplification by LA-QPCR.

To determine the effect of NEIL2 deficiency on the accumulation of oxidative DNA damage in response to SSS smoke solution, knockdown experiments were performed in cultured hPF and HEK293 cells transfected with siRNA targeting NEIL2. The efficiency of NEIL2 down-regulation was confirmed by q-RT-PCR analysis **(** **Fig. 4A** left panel) and Western blot analysis (**Fig. 4A** right panel).

**Figure 5:**
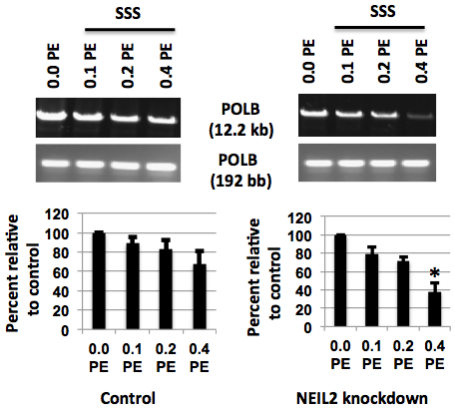
Effect of NEIL2 knockdown on induction of oxidative DNA damage by exposure to SSS for 24h in the human *POLB* gene using LA-QPCR. (A) Cultured primary hPF with NEIL2 knockdown were treated with 3 doses of SSS extract as compared to the control on the left, followed by processing of the DNA with Fpg and Nei. The DNA was used for *Polβ* (*POLB*) large and short fragment amplification by LA-QPCR. Quantification was with ImageQuant (Molecular Dynamics). The histogram depicts the mean % of DNA tail ± SEM of 3 to 4 independent experiments. The symbol * means that the differences between the Control and NEIL2 knockdown were statistically significant (P<0.05).

Down-regulation of NEIL2 in hPF led to a decrease in the amplification of the 10.4 kb *HPRT* fragment in response to SSS (**Fig. 4B** right panel) compared to those cells transfected with the control siRNA (left panel). An additional decrease in the large *HPRT* fragment was present in those fibroblasts with NEIL2 knockdown. It should be noted that in **Fig. 4B** lane 1 in the right panel (without SSS treatment), the amount of oxidative DNA damage reflects the baseline level of the damage formed endogenously as a result of NEIL2 knockdown. Similar results were also observed in HEK293 cells (**Fig. 4C**). We also confirmed significantly decreased amplification of *POLβ* (**Fig. 5**), another gene that has previously been used in laboratories to measure DNA damage [16], [22], [24]. Taken together, these results indicate a significant increase in SSS-induced oxidative DNA damage in human cells with deceased expression of NEIL2.

## Discussion

Although many DNA-adducts, particularly the bulky ones, have been identified and characterized from SHS-exposed samples, data are sparse or inconsistent on the specific oxidative DNA lesions produced by SHS in animals or non-smokers [9]. The most commonly studied lesion is 8-oxo-7,8-dihydro-2′-deoxyguanosine (8-oxo-dG), which is a widely used biomarker for oxidative DNA damage. 8-Oxo-dG mainly causes G:C to T:A transversions, which are common somatic mutations associated with several types of human cancer [26], [27]. Oxidative DNA damage was significantly increased in the lungs of rats [28] and in the lung, heart and liver of mice exposed to SSS [29]. However, there were also negative findings with regard to the corresponding oxidative DNA damage.

To the best of our knowledge, we show for the first time that SSS exposure leads to a measurable increase of oxidative DNA damage in specific genes. Using the same technique, we recently described increased levels of oxidative DNA damage in two genes of human BEAS-2B cells exposed to thirdhand smoke (THS) [22], which is consistent with the findings in this study. THS can be considered an evolved form of SHS, which undergoes chemical transformations during aging [30]. Cigarette smoking can result in mutations in a number of genes, most commonly the *P53* and *K-ras* genes, and has been implicated in the development of smoking-induced lung cancer. In these examples, the bulky acrolein- and BPDE-derived adducts are thought to correlate with the mutational spectra found in lung cancer cells [31]–[33]. It would be of interest to explore a causal relationship between oxidized DNA bases and mutations in relation to cancer, thus further improving our understanding of the mechanisms by which SHS contributes to these diseases.

As for BER, the major mechanism for the repair of oxidative DNA damage in mammalian cells, it can be mediated by multiple DNA glycosylases. [12], [34] The enzyme OGG1 primarily removes purine-derived lesions, while endonuclease III-like (NTH1) protein excises pyrimidine-derived lesions [35]. NEIL1 and NEIL2 were discovered in 2002 by Dr. Hazra and others as a family of mammalian glycosylases, which excise both purine and pyrimidine oxidation products [17], [18]. Further studies found that, unlike OGG1 and NTH1, both NEIL1 and NEIL2 can excise lesions from open DNA structures or single-stranded DNA generated transiently during DNA replication and transcription ([36], Sarker et al., unpublished data). These observations suggest that NEIL1 and NEIL2 may remove oxidized bases primarily from actively transcribing genes via transcription-coupled BER (TC-BER) and also possibly during replication. NEIL2 has been implicated in the development of cancer in certain non-lung tissues [37]. The immunohistochemical results with human lung adenocarcinomas (**Fig. 1**) suggest that lower NEIL2 levels may be associated with lung tumor development, which prompted us to investigate the effects of NEIL2 knockdown on formation of oxidative DNA damage in lung cells during SSS exposure. It was previously reported that the activity of OGG1, the primary enzyme for 8-oxoG removal, was lower in peripheral blood mononuclear cells of lung cancer patients than in controls [38]. Our data obtained with LA-QPCR, an indirect measure of oxidative DNA damage, indicate that SSS caused increased levels of such lesions in human pulmonary epithelial cells and fibroblasts and HEK293 cells with NEIL2 knockdown (**Fig. 4**). Biochemically, our data show that SSS or its derivatives induce oxidized DNA bases that fall into the substrate specificity of NEIL2, although the exact nature of such adducts are not yet known. It should also be noted that a direct comparison of the three cell lines used in our assays with regard to the levels of oxidative DNA damage is not intended, as the DNA repair and replication status in different cell lines would differentially affect the amount of damage detected in the cell.

Based on our previous data [16], NEIL2 knockdown results in an increase in spontaneous oxidative DNA damage in BEAS-2B cells. When these data are used as 100% relative to SSS-treated samples, the extent of decrease in the amplification of 10.4 kb fragment in the samples treated with 0.1 to 0.4 PE of SSS solution would just reflect the SSS-induced effect, *i.e*., an increase in oxidative DNA damage. Similar results were observed in the *POLB* gene (**Fig. 5**).

We recently showed the frequent occurrence of the R257L variant in lung cancer patients [16], Its poor repair activity might be the underlying defects causing accumulation of higher levels of endogenous oxidative DNA damage which is consistent with our current observation in which SSS exposure was the environmental challenge. In addition, our preliminary data showed that overexpression of a wild-type NEIL2 in BEAS-2B cells could rescue the NEIL2 deficiency-related phenotypic change, suggesting that reduced repair of oxidative DNA damage by an impaired NEIL2 function in non-smokers exposed to SSS, such as an NEIL2 variant, may lead to a blockage of transcription and replication. Moreover, this may result in accumulation of mutations in critical oncogenes/tumor suppressor genes of human lung cells, thus contributing to the onset of carcinogenesis.

## Supporting Information

Figure S1 **Increased level of HIF-1α in BEAS-2B cells upon exposure to SSS.** (A) Increased protein level of HIF-1α in SSS treated BEAS-2B cells as shown by Western blot analysis. GAPDH proteins were used as controls. (B) HIF-1α stabilization (changes by expression in protein level is usually a later event) by 1 mM H_2_O_2_ treatment for 60 min used as control and described previously [25].
(TIF)

Figure S2 **LA-QPCR assay with or without addition of Fpg/Nei.** (A) Both hPF and BEAS-2B cells were treated with SSS (0.4 PE) for 24 h and genomic DNA purified using the Qiagen Genomic-tip 20/G kit. The DNA was digested either with or without Fpg/Nei enzymes, and then followed by LA-QPCR amplification of the *HPRT* long and short fragments. (B) Quantification was with ImageQuant (Molecular Dynamics).
(TIF)
