# Supplementary figures and images for: NEIL2 Protects against Oxidative DNA Damage Induced by Sidestream Smoke in Human Cells
Source: PLoS One. 2014 Mar 3;9(3):e90261. doi: 10.1371/journal.pone.0090261 (PMC3945017; doi:10.1371/journal.pone.0090261)

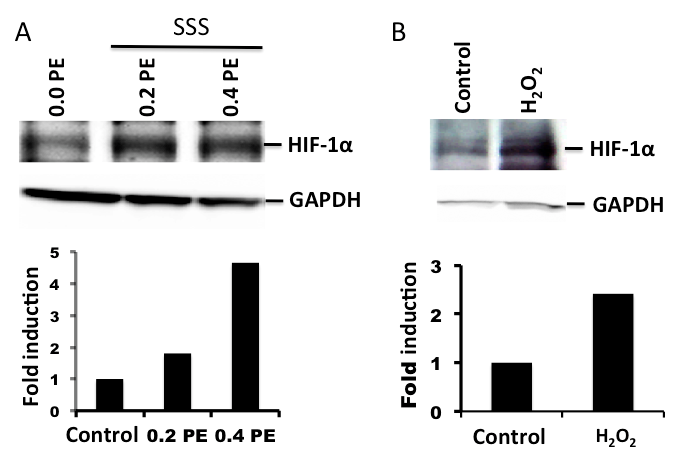

Supplement: Figure S1 — Increased level of HIF-1α in BEAS-2B cells upon exposure to SSS. (A) Increased protein level of HIF-1α in SSS treated BEAS-2B cells as shown by Western blot analysis. GAPDH proteins were used as controls. (B) HIF-1α stabilization (changes by expression in protein level is usually a later event) by 1 mM H2O2 treatment for 60 min used as control and described previously [25]. (TIF) [file pone.0090261.s001.tif]

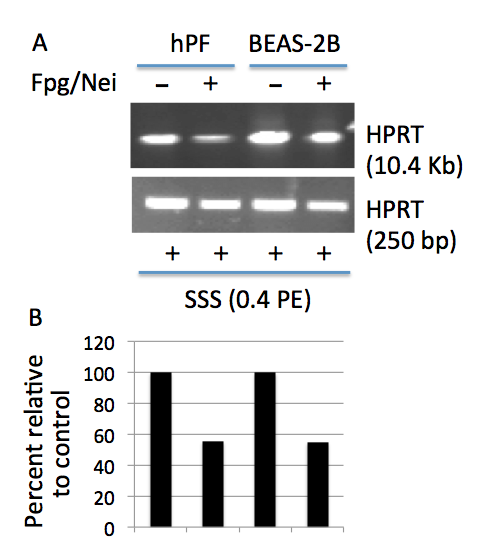

Supplement: Figure S2 — LA-QPCR assay with or without addition of Fpg/Nei. (A) Both hPF and BEAS-2B cells were treated with SSS (0.4 PE) for 24 h and genomic DNA purified using the Qiagen Genomic-tip 20/G kit. The DNA was digested either with or without Fpg/Nei enzymes, and then followed by LA-QPCR amplification of the HPRT long and short fragments. (B) Quantification was with ImageQuant (Molecular Dynamics). (TIF) [file pone.0090261.s002.tif]
